# Effect of progesterone on nitric oxide/cyclic guanosine monophosphate signaling and contraction in gastric smooth muscle cells

**DOI:** 10.3892/br.2019.1251

**Published:** 2019-11-04

**Authors:** Othman A Al-Shboul, Ayman G Mustafa, Amal Abu Omar, Ahmed N Al-Dwairi, Mohammad A Alqudah, Mona S Nazzal, Mahmoud A Alfaqih, Rami A Al-Hader

Biomed Rep 9:511-516, 2018; DOI: 10.3892/br.2018.1161

After the publication of the above paper, the second author, Ayman Mustafa, realized that his present address (Qatar University, Doha, Qatar) should have been included in the correspondence information for the manuscript. Therefore, the authors’ names and affiliations for this paper should have been presented as follows:

OTHMAN A. AL.SHBOUL^1^, AYMAN G. MUSTAFA^2,4^, AMAL ABU OMAR^2^, AHMED N. AL.DWAIRI^1^, MOHAMMAD A. ALQUDAH^1^, MONA S. NAZZAL^1^, MAHMOUD A. ALFAQIH^1^, and RAMI A. AL.HADER^3^

Departments of ^1^Physiology and Biochemistry, and ^2^Anatomy, Faculty of Medicine, Jordan University of Science and Technology, Irbid 22110; ^3^Department of Physiology and Biochemistry, Princess Basma Teaching Hospital, Faculty of Medicine, Jordan University of Science and Technology, Irbid 21110, Jordan

*Correspondence to*: Dr Othman A. Al-Shboul, Department of Physiology and Biochemistry, Faculty of Medicine, Jordan University of Science and Technology, P.O. Box 3030, Irbid 22110, Jordan

E.mail: oashboul@just.edu.jo

*Present address*: ^4^Department of Basic Medical Sciences, College of Medicine, Qatar University, Doha, Qatar

The authors regret this oversight in terms of their author affiliations, and apologize for any inconvenience caused.

